# Supplementary material for: Comparative molecular genomic analyses of a spontaneous rhesus macaque model of mismatch repair-deficient colorectal cancer
Source: PLoS Genet. 2022 Apr 21;18(4):e1010163. doi: 10.1371/journal.pgen.1010163 (PMC9064097; doi:10.1371/journal.pgen.1010163)
Supplement: S1 Table — (PDF) [file pgen.1010163.s014.pdf]

S1 Table. Comparison of human and rhesus MSI markers.

	Repeat Patterns	Rhesus MSI Markers	Repeat Patterns
**BAT 25**	$(A)_{25}$	c-kitRheBAT25	$(A)_{36}$
**BAT 26**	$(A)_{26}$	RheBAT26	$(A)_{27}$
**BAT 40**	$(T)_{7}$….$(T)_{40}$	RheBAT40	$(T)_{6}C(T)_{6}C(T)_{5}C(T)_{5}C(T)_{4}C(T)_{17}$
**D10S197**	$(CA)_{7}$…$(CA)_{12}$	RheD10S197	$(CA)_{18}$
**D18S58**	$(GC)_{5}GA(CA)_{17}$	RheD18S58	$(CA)_{18}$
**TGFβRII**	$(A)_{10}$	RheTGFβRII	$(A)_{10}$
