# Supplementary material for: Comparative molecular genomic analyses of a spontaneous rhesus macaque model of mismatch repair-deficient colorectal cancer
Source: PLoS Genet. 2022 Apr 21;18(4):e1010163. doi: 10.1371/journal.pgen.1010163 (PMC9064097; doi:10.1371/journal.pgen.1010163)
Supplement: S2 Table — (PDF) [file pgen.1010163.s015.pdf]

S2 Table. Primer sequences for determination of rhesus MSI status and *MLH1* germline mutation.

Primer ID	FW Primer (5'-3')	RV Primer (5'-3')	PCR Product (bp)	Gene
**c-kitRheBAT25**	CGAGATTGTAC CACTGCAC	TGCCTGGCTGA TATTTCTTTA	119	*c-kit*
**RheBAT26**	GATATTGCAGCA GTCAGAGC	AACCATTCAACA TTTTTAACCC	87	*MSH2*
**RheBAT40**	CCTACACCACAA TCCTGCT	GGGTGGTAGAGC AAGACCT	144	*3β-HSD*
**RheD10S197**	CTTCAGGGTGAAA GGACGAG	ACCTCGAGTGGCA TTTTGAA	128	*GAD2*
**RheD18S58**	TCCCTTAGGAGGC AGGAAAT	TCCTGGCCGGCTT TATTTAT	153	*DCC*
**RheTGFβRII**	TGACTTTATTCTGG AAGATGCTG	AACACATGAAGAA AGTCTCACCA	83	*TGF-β-RII*
**RheMLH1**	TAACAGGCAAAAAT CTGGGC	CCACATACACCATA TGTGCC	324	*MLH1*
**RheBRAF**	CCTAAAATCTTCAT AATGCTT	ATAGCCTCAATTCT TACCAT	209	*BRAF*
