# Supplementary material for: Comparative molecular genomic analyses of a spontaneous rhesus macaque model of mismatch repair-deficient colorectal cancer
Source: PLoS Genet. 2022 Apr 21;18(4):e1010163. doi: 10.1371/journal.pgen.1010163 (PMC9064097; doi:10.1371/journal.pgen.1010163)
Supplement: S3 Table — This analysis was performed using a random forest classifier in the CMSclassifier (R studio) to establish CMS status in rhesus CRC. CMS calls are indicated for each of the samples in bold. (PDF) [file pgen.1010163.s016.pdf]

S3 Table. CMS classification of rhesus CRC. This analysis was performed using a random forest classifier in the CMSclassifier (R studio) to establish CMS status in rhesus CRC. CMS calls are indicated for each of the samples in bold.

Sample	CMS1	CMS2	CMS3	CMS4	Predicted CMS (RF)	MSI status
RM18	0.25	**0.61**	0.12	0.02	CMS2	MSI-H
RM13	0.36	**0.42**	0.09	0.13	CMS2	MSI-H
RM14	0.20	**0.40**	0.13	0.27	CMS2	MSI-H
RM04	0.21	**0.37**	0.31	0.11	CMS2	MSI-L
RM01	0.22	0.26	0.13	**0.39**	CMS4	MSI-L
RM02	0.12	**0.60**	0.17	0.11	CMS2	MSS
RM08	0.33	**0.39**	0.24	0.04	CMS2	MSI-H
RM17	0.14	0.12	0.03	**0.71**	CMS4	MSI-L
RM09	**0.36**	0.32	0.26	0.06	CMS1	MSI-H
RM26	0.25	**0.55**	0.18	0.02	CMS2	MSS
RM33	**0.42**	0.32	0.19	0.07	CMS1	MSI-H
RM23	**0.35**	0.31	0.08	0.26	CMS1	MSI-H
RM27	0.28	**0.42**	0.28	0.02	CMS2	MSI-H
RM40	0.14	0.22	0.10	**0.54**	CMS4	MSI-H
RM30	0.24	**0.48**	0.23	0.05	CMS2	MSI-H
RM37	**0.42**	**0.42**	0.13	0.03	CMS1-CMS2	MSI-H
RM31	0.24	**0.63**	0.10	0.03	CMS2	MSI-H
RM38	**0.44**	0.22	0.21	0.13	CMS1	MSI-H
RM25	0.29	0.25	0.12	**0.34**	CMS4	MSI-H
