# Supplementary material for: Comparative molecular genomic analyses of a spontaneous rhesus macaque model of mismatch repair-deficient colorectal cancer
Source: PLoS Genet. 2022 Apr 21;18(4):e1010163. doi: 10.1371/journal.pgen.1010163 (PMC9064097; doi:10.1371/journal.pgen.1010163)
Supplement: S4 Table — Total RNA sequencing was performed in 19 tumor and 16 matched normal samples. DNA methylation using RRBS was performed in 14 tumor and 14 matched normal tissues. Methylation analysis of MLH1 was performed in 7 tumor and 3 normal samples (2 matched). Samples profiled with more than one platform are marked with a blue background. (PDF) [file pgen.1010163.s017.pdf]

S4 Table. Summary of genomic analyses performed in specimens from *Rhesus macaques* presented in this manuscript. Total RNA sequencing was performed in 19 tumor and 16 matched normal samples. DNA methylation using RRBS was performed in 14 tumor and 14 matched normal tissues. Methylation analysis of *MLH1* was performed in 7 tumor and 3 normal samples (2 matched). Samples profiled with more than one platform are marked with a blue background.

	Total RNA seq			RRBS			*MLH1* DNA methylation		
**Animal ID**	**Batch**	**Sequencing Platform**	**Sample Type**	**Batch**	**Sequencing Platform**	**Sample Type**	**Batch**	**Sequencing Platform**	**Sample Type**
RM1	1	HiSeq-4000	T	1	NovaSeq-6000	T/N	-	-	-
RM2	1	HiSeq-4000	T/N	1	NovaSeq-6000	T/N	-	-	-
RM3	-	-	-	-	-	-	-	-	-
RM4	1	HiSeq-4000	T/N	-	-	-	-	-	-
RM5	-	-	-	-	-	-	-	-	-
RM6	-	-	-	-	-	-	1	Ion Torrent	T
RM7	-	-	-	-	-	-	-	-	-
RM8	1	HiSeq-4000	T/N	-	-	-	1	Ion Torrent	T
RM9	2	HiSeq-4000	T	-	-	-	1	Ion Torrent	T
RM10	-	-	-	1	NovaSeq-6000	T/N	1	Ion Torrent	T
RM11	-	-	-	-	-	-	1	Ion Torrent	N
RM12	-	-	-	-	-	-	-	-	-
RM13	1	HiSeq-4000	T	-	-	-	1	Ion Torrent	T/N
RM14	1	HiSeq-4000	T/N	-	-	-	1	Ion Torrent	T
RM15	-	-	-	-	-	-	1	Ion Torrent	T/N
RM16	-	-	-	-	-	-	-	-	-
RM17	2	HiSeq-4000	T/N	1	NovaSeq-6000	T/N	-	-	-
RM18	1	HiSeq-4000	T/N	1	NovaSeq-6000	T/N	-	-	-
RM19	-	-	-	-	-	-	-	-	-
RM20	-	-	-	-	-	-	-	-	-
RM21	-	-	-	-	-	-	-	-	-
RM22	-	-	-	-	-	-	-	-	-
RM23	3	NovaSeq-6000	T/N	1	NovaSeq-6000	T/N	-	-	-
RM24	-	-	-	-	-	-	-	-	-

RM25	3	NovaSeq-6000	T/N	1	NovaSeq-6000	T/N	-	-	-
RM26	3	NovaSeq-6000	T/N	1	NovaSeq-6000	T/N	-	-	-
RM27	3	NovaSeq-6000	T/N	-	-	-	-	-	-
RM28	-	-	-	-	-	-	-	-	-
RM29	-	-	-	-	-	-	-	-	-
RM30	3	NovaSeq-6000	T/N	1	NovaSeq-6000	T/N	-	-	-
RM31	3	NovaSeq-6000	T/N	1	NovaSeq-6000	T/N	-	-	-
RM32	-	-	-	-	-	-	-	-	-
RM33	3	NovaSeq-6000	T/N	1	NovaSeq-6000	T/N	-	-	-
RM34	-	-	-	-	-	-	-	-	-
RM35	-	-	-	-	-	-	-	-	-
RM36	-	-	-	-	-	-	-	-	-
RM37	3	NovaSeq-6000	T/N	1	NovaSeq-6000	T/N	-	-	-
RM38	3	NovaSeq-6000	T/N	1	NovaSeq-6000	T/N	-	-	-
RM39	-	-	-	-	-	-	-	-	-
RM40	3	NovaSeq-6000	T/N	1	NovaSeq-6000	T/N	-	-	-
RM41	-	-	-	-	-	-	-	-	-
