# Supplementary material for: Comparative molecular genomic analyses of a spontaneous rhesus macaque model of mismatch repair-deficient colorectal cancer
Source: PLoS Genet. 2022 Apr 21;18(4):e1010163. doi: 10.1371/journal.pgen.1010163 (PMC9064097; doi:10.1371/journal.pgen.1010163)
Supplement: Attachment — Submitted filename: PGENETICS-D-21-01125_Rebuttal_Letter.docx [file pgen.1010163.s018.docx]

January 22, 2022

Carlos E. Alvarez, PhD

**Guest Editor**

PLOS Genetics

Dear Dr. Alvarez,

We are pleased to submit a revised version of the manuscript PGENETICS-D-21-01125 titled *‘Comparative Molecular Genomic Analysis of a Spontaneous Rhesus Macaque Model of Mismatch Repair-Deficient Colorectal Cancer’* to be considered for publication in *PLOS Genetics*.

We thank you and the Reviewers for their comments and extremely constructive suggestions. We feel that the revised manuscript is now addressing the concerns raised by the Reviewers. We have made a great effort to incorporate these suggested modifications at the same time that we have tried to keep the word count as close as possible to the recommended word limit.

Responses to the Reviewer’s comments are provided below in *italics* with references to manuscript page and line numbers. Please, note that page and line calls within this Rebuttal Letter correspond to the clean version (non-tracked) of the manuscript file. In addition, we have attached a marked version of the previous manuscript to highlight the changes for your and Reviewer’s reference. Following your instructions, the current version adheres strictly to all the formatting requirements provided in the decision letter.

**Reviewer #1** - This is a highly interesting description of a rhesus macaque model where some developed MLH1 deficient colorectal cancers either due to sporadic MLH1 hypermethylation or due to germline MLH1 mutations conferring a rhesus macaque version of Lynch syndrome. The authors have demonstrated the molecular basis of the 41 cases of colorectal cancer and have developed an MSI panel more specific for the rhesus macaque model. In trying to describe the rhesus macaque MLH1 Lynch syndrome animal model, I believe there are some additional details that would help clarify and characterize the authors argument that this would be the preferred animal model of study.

*We thank the Reviewer for the enthusiastic support of our study and appreciate the opportunity to strengthen our findings. We believe this revised version of our manuscript addresses the Reviewer’s comments and recommendations and incorporates additional information that helps clarify the characterization of the Lynch Syndrome Rhesus model.*

1. Are there any general details about the colony and what is the overall prevalence of colorectal cancer? Is the prevalence similar to that of ~6% of colorectal cancer in the general population?

*To help clarify the general details of the colony and the overall prevalence of CRC, we have added the following text in the Material and Methods subsection of the manuscript (page 18, lines 419-436): ‘The rhesus macaque breeding colony, called the Rhesus Monkey Breeding Research Resource (RMBRR), was established as a SPF colony in 1989. Establishment of the colony began in 1974-1975 with 17 male and 74 female Indian-origin rhesus macaques. The colony has remained SPF since 1989 and has been closed since its founding. Mating schema is harem breeding with one male and generally 3 to 12 females in a social breeding group. Kinship coefficients of all males and females are screened prior to assembling breeding groups to minimize inbreeding. Current practice is to avoid mating between animals with kinship coefficients >0.007. For reference, two 2nd cousins have a kinship coefficient of 0.0156, and two 3rd cousins have 0.00391. Environmental conditions, including diet and behavioral enrichment are comparable across all animals in the colony and have not changed significantly over time.’*

*‘The first case of CRC in the RMBRR was diagnosed 1988 in one of the founding males. The exact age of this animal is not known but estimated to be >15 years. The allele frequency of the MLH1 mutation within the RMBRR is currently approximately 5%. MLH1 carriers have not been segregate in the population. Cumulative prevalence of CRC in the RMBRR in the period 2003-2019 is: 0.11% for ages 8-12 years; 1.68% for 13-17 years; 5.22% for 18-22 years, and 10.17% for 23-27 years.’*

2. Could the authors provide detailed pedigrees of the macaque models where germline MLH1 pathogenic variants were discovered? Were there clusters of affected animals that are related to each other over several generations? Does the transmission suggest an autosomal dominant transmission of susceptibility?

*We agree with reviewer #1 that providing a detailed pedigree will help the readership understanding the heritability of the pathogenic germline MLH1 mutation. We are now adding a detailed pedigree in S3 Fig. In addition, we have added the following text to the Colony demographic subsection under Materials and methods (page 19, line 434-436): ‘Rhesus MLH1 carriers with CRC reported in this paper demonstrate inheritance patterns consistent with autosomal dominance with incomplete penetrance (S3 Fig).’ Although we recognize that no animal model will exactly recapitulate the human disease, it is interesting to note that the cohort of MLH1 carriers (rhesus Lynch Syndrome) reported in our manuscript appears to meet the “3-2-1 rule” of the Revised Bethesda guidelines to identify individuals with CRC who should undergo testing for MSI for Lynch Syndrome and sporadic MSI-H diagnosis. The 3-2-1 rule refers to at least 3 affected family members, over at least 2 generations, with at least 1 affected under 50 years of age. An age for a rhesus analogous to 50 years in humans would be 16-17 years given that the typical lifespan of a captive rhesus macaque is 25-30 years. As seen in* ***S3 Fig****, family inheritance patterns associated with RM17 and RM9 appear to fit the 3-2-1 rule with RM17 and RM9 being aged 11 and 15 years respectively. We have incorporated text to illustrate this point on Page 8, Lines 161-164 and added the original Reference of the Revised Bethesda criteria (Reference #13).*

3. Were there other any other cancers found in the germline MLH1 macaque carriers like those found in human Lynch syndrome families? For example, were there any other animals with Lynch associated cancers (endometrial, ovarian, urothelial)?

*As illustrated in S3 Fig, RM Y, a half-sibling to RM25 and RM9 developed a slow growing salivary adenocarcinoma.*

4. The authors should also provide a figure of the pathologic IHC stained sections that demonstrate loss of MLH1 as well as the staining of MSH2, MSH6, and PMS2. What would also be interesting to check if high tumor infiltrating lymphocytes (TIL) counts (seen in human Lynch syndrome CRC tumors) were found in the germline MLH1 deficient tumors?

*Thank you to Reviewer #1 for this excellent suggestion. We have added images of IHC staining for MLH1, MSH2, MSH6, and PMS2 from rhesus CRC tissue samples (S4 Fig). In this figure, we have included sections of control colorectal mucosa (no tumor), MSS tumor, and MSH-H tumor slides. Slide images are presented at 100X magnification and the image is referenced in the text on page 9, line 170-171. In addition, our group has previously published the IHC characterization of an independent cohort of this model noted in Reference #5 and #10. In addition, tumor-infiltraiting lymphoyctes are commonly observed in these tumors, which has been prevsioly reported by our group as noted in Reference #10.*

5. Could the authors discuss the feasibility of creating a macaque model of MSH2, MSH6, PMS, and EPCAM germline mutations?

*Thank you to Reviewer #1 for the opportunity to elaborate on this interesting and trending topic in primate research. We report here a novel spontaneous NHP model for MLH1-deficient Lynch Syndrome and more generally sporadic CRC. An important context to this discussion is to summarize and contrast the different advantages that an outbred spontaneous NHP model and inbred genetically engineered mouse models (GEMMs) offer. GEMMs offer a lower cost animal model with a short reproduction and multiple offspring per litter whereby -generally speaking- the effect of loss or gain or altered function of individual gene(s) can be assessed against a relatively homogeneous genetic background, given that the vast majority of GEMMs are inbred. Without question, GEMMs provide efficiencies and avenues to study gene function that is not comparably possible in other animal models. In contrast, NHP spontaneous models of complex diseases do not offer the advantages of GEMMs to study gene(s) in relative isolation because the genetic background is not inbred, but rather is heterogeneous. However, pedigreed NHP breeding colonies offer avenues to study the relative contribution of genes to complex disease that are not comparably possible with either GEMMs or human populations. This is due to the ability to maintain a high level of uniformity of environmental conditions in NHP colonies while also knowing the degree of genetic relatedness between individuals.*

*Gene editing technologies such as CRISPR/Cas9 seem to have enormous potential to develop novel biomedical research models. NHP models have not been widely used for such gene editing technologies in biomedical research at the present time, but that is likely to change in the next decade or sooner. As that moves forward it will be important to be cognizant of the advantages and limitations GEMMs, outbred spontaneous models, pedigreed NHP colonies, and any future gene-edited NHPs each offer. For example, in contemplating the future possibility, feasibility, and value of creating targeted gene editing of MLH1, MSH2, MSH6, PMS, or EPCAM in a NHP with the intent to model MMR-deficient human Lynch Syndrome, one should consider the expected length of time for onset for the disease phenotype. Other considerations should include the relative penetrance seen in human LS with each of the MMR genes. MSH6 and PMS2 lead to phenotypes that are less penetrant in humans. MSH2 is as penetrant as MLH1 in humans but more frequently leads to ovarian or endometrial cancer. Such considerations should be balanced a priori against what questions would be expected to be answered with a gene-edited NHP that could not be potentially answered by a spontaneous model. It should be noted that other NHP species with shorter gestation times and more than one offspring per parturition event (e.g. marmosets) will likely be developed as CRISPR/Cas9 technologies continue to be utilized in NHPs. To conclude, when and if NHPs do become more widely used gene-edited models of complex disease, such a development will likely augment rather than replace the need and value of spontaneous NHP models within pedigreed colonies given the interaction of genes and environment for disease phenotype expression.*

*We have summarized this comment and added one paragraph to the discussion section (Page 17, lines 370-384).*

**Reviewer #2** - The authors report comprehensive molecular characterization of colorectal tumors associated with a germline mutation in the MLH1 gene in Rhesus macaque. The study reveals a number of similarities between these non-human tumors and human colorectal tumors. The work is interesting. However, what types of preclinical and clinical research can be better conducted with the Rhesus model, compared to directly studying human CRC? What about the cost? Furthermore, a number of technical issues need to be addressed

*We thank Reviewer #2 for the acknowledgement of our interesting findings and recognize the need to clarify several issues that were not clear in the initial version of the manuscript. We greatly appreciate this opportunity to revise the manuscript.*

1. “CMS classification categorized rhesus CRC samples mainly as CMS2”: why are they mainly CMS2? Should the Rhesus tumors of MSI-H be classified as CMS1, like in humans?

*We agree with Reviewer #2 that Rhesus tumors would presumably classify as CMS1 as observed in human cases. In the discussion section, we have further elaborated our interpretation of the CMS classification results in the rhesus model (Page 16, lines 340-344, and page 17, lines 345-353). Briefly, one possbiblity may be that tumor stage at diagnosis in rhesus was far more advanced than typically seen in humans where surveillance colonoscopy is standard of care and a very frequent method of diagnosis. Therefore, detection at different stages between humans and rhesus may help explain the CMS differences.*

2. Mutation burden: MSI-H colorectal tumors in human are characterized with high mutation burden; is the same observed in rhesus colorectal tumors?

*We thank Reviewer #2 for suggesting to add the tumor mutation burden (TMB) assessment to our manuscript. We have added the rate of mutations/Mb in conding regions per sample (mutational load) to S11 Fig and added a note into the text (Page 13, lines 283-284). These rates are moderately high in some of the samples, but we aknowledge they are not comparable to the threshold established in human tumors to indicate hypermutation (>10 mutations/Mb). However, our assessment of TMB may be biased as it has been performed using total RNAseq, which is not the most optimal way to establish mutational burden being the gold standard whole exome sequencing.*

3. The integration between DNA methylation and gene expression should be conducted? E.g., will promoter methylation lead to decrease in gene expression in what genes?

*We agree with Reviewer #2 that understanding the relationship between DNA methylation and gene expression levels is important and needed in this study. We have added additional data reporting the correlation analysis between DNA methylation and gene expression (S7B Fig). The data suggests a trend towards a significant negative correlation between DNA methylation and gene expression. We are now presenting this information in the revised manuscript (Pages 10-11, lines 219-222.)*

4. Figure 3A: PC1+PC2 is only around 25%, too small for a meaningful interpretation. The same is true for Figure 4A. 3D PCA or TSNE may be used.

*We agree with Reviewer #2 that a 3D PCA plot is needed to glean a meaningful interpretation of the presented data. We have changed both Fig 3A and 4A to 3-D PCA plots.*

5. Figure 3B: no p-values indicating how stable the clusters are.

*We have provided an additional figure (S6 Fig) that demonstrates cluster stability by including the approximately unbiased (au) p-values and bootstrap probabililty (bp) values for the data presented in Fig 3B. We have also added a comment within the text (Page 10, line 207-208).*

6. More thorough comparison of each finding with that of human CRC is needed.

*We acknowledge the need for further cross-comparison between human and rhesus CRC to strengthen the findings in our study. We have added supplemental data on genomic characterization (S11 and S12 Figs) with an special emphasis on presenting mutations in commonly altered genes in human CRC that were also observed in rhesus (Page 13, line 282-291). In addition, we have significantly expanded the discussion section and offered comparisons of the results of every layer of the genomic characterization with references to the human picture for MSI analysis (Page 15, lines 319-323), methylation (Page 15, lines 328-330), transcriptomics (Page 15, lines 335-344), pathways enrichment and CMS subclassification (Page 16, lines 345-353), and mutations (Page 16, lines 362-368).*

**Reviewer #3 (Dr. Modiano)** - The authors describe the mismatch repair (MMR) status of spontaneous colorectal carcinoma (CRC) in 41 animals from a closed Rhesus macaque colony at MDACC. The results are intriguing and have the potential to add considerable value to the extant information of MMR in the pathogenesis of CRC.

The data are mostly clear, with exceptions noted below.

However, critical aspects of the experiments and analysis are omitted, and the enthusiasm for the manuscript in its current form is diminished because the authors vastly over-interpret the significance and potential of what are admittedly important data, but in a model system with many more limitations than the authors acknowledge.

Specific issues of concern are enumerated below:

*We thank Dr. Modiano for giving us the opportunity to appropriately contextualize the findings of our work. We believe our revised version encompasses the specifc issues of concerns raised by Reviewer #3, and we have made an effort to tone down our interpretation to better align with the data at hand.*

1. Even though the authors provide background into the colony and the presence of a group with the spontaneous MLH1 (c.1029C<G, p.Tyr343Ter) mutation, it is challenging to interpret the data without a denominator. The authors examine samples from 41 animals. But there is no reference as to the timepoint over which these 41 animals were collected, how large the colony was, and the proportion and pedigree of animals harboring the mutation in the colony.

*To help clarify the general details of the colony and the overall prevalence of CRC, we have added the following text as a Material and Methods subsection in the manuscript (page 19, lines 419-427): ‘The rhesus macaque breeding colony, called the Rhesus Monkey Breeding Research Resource (RMBRR), was established as a SPF colony in 1989. Establishment of the colony began in 1974-1975 with 17 male and 74 female Indian-origin rhesus macaques. The colony has remained SPF since 1989 and has been closed since its founding. Mating schema is harem breeding with one male and generally 3 to 12 females in a social breeding group. Kinship coefficients of all males and females are screened prior to assembling breeding groups to minimize inbreeding. Current practice is to avoid mating between animals with kinship coefficients >0.007. For reference, two 2nd cousins have a kinship coefficient of 0.0156, and two 3rd cousins have 0.00391. Environmental conditions, including diet and behavioral enrichment are comparable across all animals in the colony and have not changed significantly over time.’*

*Additional details are provided on Page 20, lines 430-434: ‘The first case of CRC in the RMBRR was diagnosed 1988 in one of the founding males. The exact age of this animal is not known but estimated to be >15 years. The allele frequency of the MLH1 mutation within the RMBRR is currently approximately 5%. MLH1 carriers have not been segregate in the population. Cumulative prevalence of CRC in the RMBRR in the period 2003-2019 is: 0.11% for ages 8-12 years; 1.68% for 13-17 years; 5.22% for 18-22 years, and 10.17% for 23-27 years.’*

*The RMBRR colony size has grown since its founding and is now maintained at an overall census of approximately 1000. Although one of the founder males developed the first recorded case of CRC, multiple and consistent cases of CRC in the population did not present until the population had aged sufficiently to have cohorts of animals >9 years of age. For example, the oldest animals in the colony were approximately 15 years of age in 2001, 20 years in 2006, 25 years in 2011, and 29 years currently in 2022.*

*Addtionally, we have added a detailed pedigree (S3 Fig) to help visualize the transmission and inheritance pattern of the germline MLH1 mutation in the macaque model and added an specific comment on the pedigree in Page 20, lines 434-436.*

2. Importantly, have the animals with the mutation been segregated? Are the environmental conditions, including diet and behavioral enrichment comparable, and have they changed over time? Is there an increasing incidence of CRC in the animals harboring the mutation over time (what is the penetrance and how many animals with the mutation develop CRC or other cancers over their lifetime) and in the population that retains wild type MLH1 in both maternal and paternal alleles?

*We thank Dr. Modiano for asking this important question and for the opportunity to incorporate this relevant information to the readership of our manuscript. MLH1 carriers have not been segregated in the population (now stated in Page 20, line 432-433). Environmental conditions, including diet and behavioral enrichment are comparable across all animals in the colony and have not changed significantly over time (now stated in Page 19, lines 426-428). Pedigreed NHP populations with their relative uniformity of environmental conditions and known genetic relationships represent a unique research resource for studies attempting to delineate genetic contribution to complex diseases in a way that is not possible in inbred models (such as rodents) or in human populations where environmental conditions are highly variable and genetic relationships and/or admixture is not known (this is now discussed on Page 17, lines 370-375). The rhesus MLH1 carriers with rhesus-Lynch/CRC reported in this paper demonstrate inheritance patterns consistent with autosomal dominance with incomplete penetrance (S3 Fig, Page 20, lines 434-436; Page 8, lines 161-164 and new Reference #13; Please, see also our reply to Reviewer #1, point #2). Finally, comprehensive retrospective genotyping of all deceased rhesus with archived tissue within the RMBRR is ongoing and part of a larger project which will eventually allow a sufficiently robust calculation of penetrance. In the meantime, we are now providing prevalence calculations as reflected in the previous reply to point #1 and now included in Page 20, lines 433-436.*

3. Is there evidence of imprinting (greater frequency or shorter lag time of disease in animals that inherit a paternal vs a maternal allele)? As a reviewer who is often on the other side (author), I realize that these are not trivial requests, but the scale and magnitude of the effect and the impact of the mutation on the disease are critical aspects necessary to assess the significance of the model and to justify more than superficial curiosity of what would otherwise be an expected result (inactivation of MLH1 leading to CRC). It would be acceptable to include this information as Supplementary materials, and preferably not as a separate manuscript, which places the burden of finding this important information on the reader.

*Rhesus MLH1 carriers with CRC reported in this paper demonstrate inheritance patterns consistent with autosomal dominance with incomplete penetrance (S3 Fig, and comments in Page 20, lines 434-436). Retrospective genotyping for the germline MLH1 mutation from DNA extracted from archived formalin-fixed tissue from a more comprehensive cohort in this NHP colony represents a monumental ongoing effort that is being performed by co-author Dr. Stanton Gray. Upon completion of this effort, the data will allow a more thorough delineation of inheritance of the MLH1 mutation, including calculation of penetrance, lifetime risk, prevalence, and potential assessment of the possible differences in the phenotype and/or age at presentation of CRC between maternally and paternally inherited MLH1 mutant alleles. Such assessments are beyond the scope of the current work, but do represent our next steps in the shortest term. We acknowledge the important value of the request made by Dr. Modiano and appreciate the suggestion to incorporate the data as supplement. However, we feel that this would add another manuscript within the current manuscript. We are committed to the publication of this data in an upcoming separate piece.*

4. It seems likely that these samples were collected over a time period (which must be stated) and that the technology used to obtain the data for analysis changed over time. For example, the reviewers refer to sequencing in two different instruments. Yet, the methods do not describe batch effects and the steps taken to achieve batch correction. This is essential to interpret the methylation and the RNA sequencing data. Depending on which samples were sequenced in which instrument at which time, batch effects could be driving much of the separation along principal components and for the differentially expressed genes (DEGs). This could explain, for example, the gene expression data where MSS/MSI-L samples are indistinguishable from MSI-H samples based on hierarchical clustering using the top DEGs (Fig 4C). The methods should include a section describing the time course of sample acquisition, processing, and sequencing, as well as batch effects and methods for batch correction. Visual representation of the batch effects and the correction should be included as supplementary materials.

*We apologize to Dr. Modiano for any confusion and agree that the sample collection time period needs to be stated. Samples were collected at necropsy between 2008 and 2019 (we are now including this information in Page 19, line 415), and FFPE blocks were prepared right after sample collection. DNA and RNA extractions were completed using AllPrep DNA/RNA FFPE Kit and, extracted DNA/RNA were kept at -20/-80 °C (we are now providing details on Page 20, lines 438-449). We have added an additional table (S4 Table) containing all information about samples analyzed per animal (tumor and normal), batches, and platforms that were used in the experiments described in the manuscript. For PCA of RNA sequencing, batch effects were removed using limma using the removeBatchEffect tool. As requested by Dr. Modiano, Fig 4A includes a 3D PCA with batch effect correction, and we are now adding a new figure (S10A Fig) displaying a 3D PCA without batch effect correction as requested. Both Figures rendered similar outcomes.*

5. The presentation of the animals that have LS (mutant MLH1) and those that inactivate MLH1 sporadically by methylation is quite confusing. Specifically, there were 8 animals with LS. Despite the authors suggestion from Fig. 3A that these tumors segregate as a group based on their methylation status, the clustering in Fig 3C actually shows that the 4 LS animals that were analyzed (presumably the samples from the other 4 animals were not of sufficient quality or there was a process of selection that should be described) do not cluster as a group. Two samples cluster with MSI-H samples and the other two cluster with a heterogeneous group of normal and MSS/MSI-L tumors as a sub-branch of the group that includes all the normal samples. There is a clear branch that includes all the MSI-H samples and two LS samples. So, based on this, Fig 3C is a non sequitur, since the authors analyze methylation differences for all the tumors together vs. normal, when the unsupervised data already distinguish a difference between MSI-H + 2 LS vs all the other samples. The fact that all tumors have hypermethylation of TOP1, PCGF3, FAM76B, and others, and hypomethylation of ALKBH5, GAS8, MME, and many others may simply be a consequence of proliferation or the generation of a TME and is not deeply informative for the major thrust of the paper, which is describing the impact of MMR and the potential utility of the model.

*We apologize to Dr. Modiano for the confusion generated in our initial submission. We realized that we have not explained to the audience that rhesus LS CRC can display a MSI-H or a MSI-L/MSS status. Although, the most frequent scenario is that CRC tumors arising in rhesus LS animals are MSI-H, MSI-L/MSS patterns are also possible, although unfrequent. We have clarified the intersection beyween MSI-H/MSI-L/MSS status and LS/sporadic tumors in Page 9, lines 189-194. We are now adding a paragraph in the discussion section to elaborate on this observation and provide a reference in the human literature to illustrate this point, which also highlights that the rhesus model recapitulates this feature observed in humans (Page 15, lines 319-323). We feel that this should help the readership to understand the actual biological context of the methylation results. We have also made our data more clear in regards to the LS/sporadic and MSI subtype status within the figures by adding a covariate bar in Fig 4C that allows to clearly compare the overlap between both subclassifications.*

*To address the important point brought by Dr. Modiano, we have now included comparisons between MSI-H (sporadic and LS) with MSI-L/MSS (sporadic and LS) tumors to identify methylation differences. To enhance the clarity, prove the rigor of our analysis, and better illustrate the relative distance between the methylation branching and the groups, we now show in a new figure (S6 Fig), P-values of correlations between samples and the actual physical distance of them with values for the analysis. The entire section of methylation results have been edited and there is new additional text illustrating all these analysis for full disclosure and transparency (See Page 10 and 11, lines 196-222). Please, note that PK1B, B4GALT7, GPR35, MYT1L and CYB5D2 are among hyper-methylated genes, and GRB10, SOD1, TMSB10 and CD52 are hypo-methylated. In addition, we present in a separate analysis methylation differences between normal and tumor samples along with correlations between methylation and expression changes as requested as well by Reviewer #2 in point #3 (See S7A and S7B Fig). Finally, to better represent group clustering in the principal compent analyses, we have added 3D PCA plots (Fig 3A and 4A), which we believe help glean a more meaningful interpretation of the presented data.*

*We realized that gene expression and methylation analysis needed to be further elaborated and we are grateful to Reviewer #2 and Dr. Modiano for their comments and the opportunity to significantly enhance the quality of the presentation of our data and the clarity.*

6. The same applies to Fig 4, where the LS animals (how many??) are simply grouped with MSI-H and not identified separately (panel A and panel C), but they are used to compare against the TCGA data from human colon and rectal carcinomas. This seems to be inappropriate data selection. Further on Figure 4, the authors neglect to describe the data in panel C in the manuscript, although there is a reference to Fig 4D, which is not provided. Perhaps that is a typo? Although, the authors describe a defined LS cohort based on gene expression, and this is definitely not apparent (or identified by legend or color code) in Fig 4. Simply, the authors description/interpretation of the data (text) is inconsistent with the data shown in the figure (Fig 4).

*We thank Dr. Modiano for this comments, and we realized that this comment goes hand in hand with the previous comment (point #5). We apologize for the confusion generated. In our revised edition, we have included a type bar to the heatmap (Fig 4C) to display the status regarding LS, sporadic MSI-H, sporadic MSI-L/MSS samples. We apoligze for negating to reference Fig 4C in our original version. We have updated the text with a description of panel C (Page 12, lines 253-256).*

7. The point above (#4) illustrates the confusion about the segregation of animals with LS and those animals with sporadic MSI-H. It seems that the tumors in the animals with LS diverge in their biology (Fig 2F and Fig 3B), perhaps less so than the sporadic MSI-H tumors, but the authors split and lump these together in the analysis and in the discussion. As a specific example, in Fig 2F the authors do not segregate the LS animals from the MSI-H animals in the description of “sporadic,” as the addition of 31 MSI-H (referred to as sporadic MSI) + 6 MSI-L + 4 MSS = 41, and which means the LS animals (which would not, or should not be considered as “sporadic,” but rather should be considered as “familial” or “heritable,” are lumped into MSI-H (sporadic) or MSI-L categories.

*We apologize for the confusion regarding segregation of animals with LS and those with sporadic MSI-H CRC. We see that this is a recurrent theme raised by Dr. Modiano in points #5, #6 and #7. Please, refer to our first paragraph in point #5 for a full explanation. In brief, PCR based MSI testing results were analyzed based on the presence of microsatellite instability. Samples were labeled as MSI-H, MSI-L or MSS by calculation the instable markers as mentioned in the Materials and Methods (Page 21, line 477-479). LS can show MSI-H or MSI-L phenotype, and our cohort had 2 LS animals with MSI-L phenotype and 6 LS animals with MSI-H phenotype (Fig 2C and 2F) and also referenced on page 9, lines 191-194. We also explain this fact in the context of methylation (Page 10, lines gene expression analysis in Page 202-213) and global transcriptomics (Page 12, lines 255-256)*

8. Suppl Table 3, in particular, shows that one of the LS animals that was used to characterize the subtype of CRC was MSI-L (RM 17, acknowledged by the authors) and two were MSI-H (RM09 and RM31). RM11 also has an MSI-L phenotype (Fig 2). It seems that the same animals were not used across the analyses shown. For example, RM09 is not included in Fig. 3. It is unclear which animals are included in the analysis of Fig. 5. All of this makes Fig. 1 disingenuous, as the impression is that all animals were used for all of the analysis, except for technical exclusions.

*We apologize to Dr. Modiano for the disingenuous impression that we have provided in the initial version of our manuscript. This is very far from our intention, which is to fullly disclose all the data and analysis for the benefit of the scientific community. In fact, we have already provided access to the gene expression files that have been deposited in GEO for re-analysis by independent computational scientists (Page 2, section on availability of data and materials). Following Dr. Modiano’s suggestions, we are now displaying in Fig 1 specific numbers of samples (tumor and normal) for each of the analysis performed in this manuscript. In addition, we have added a supplemental table (S4 Table) that details information of samples studied per animal, type of sample (tumor, normal), and platform used as well as the number of batches for each experiement. Dr. Modiano is correct that we have not performed all genomic assessments in all the samples (whole transcriptomics, MLH1 methylation and RRBS). However, we have performed IHC of MMR proteins and MSI by PCR in all the samples described in this cohort. We hope that the changes highlighted in this reply demonstrate our commitment to provide the highest levels of rigor and reproducibility to our analysis of all animal experiments.*

9. The authors acknowledge that RNAseq is not the best method to identify mutations, although the point out it is "adequate." This is true when seeking to identify mutations in specific genes (rather than globally). But to make the data interpretable, the authors should include tumor content in the sample, presumed enrichment from macro-dissection, and the number of transcripts from variant alleles vs the number of transcripts from all alleles (VA/VA+WTA). Together, this information will help estimate the VAF in the tumor and provide assurance that the presumed mutations are not sequencing artifacts. Another relatively simple method to confirm the RNAseq data would be to select a random (statistically validated) sample set where mutations were identified and perform Sanger sequencing of DNA from tumor and normal for the specific region identified.

*We agree with Dr. Modiano that adding quantification of tumor content provides assurance that the presumed mutations are not sequencing artifcats. To this point, we have calculated tumor purities using tumor and normal gene expression data using the ISOpureR package (see Material and Methods section, Page 25, line 572). The average tumor purity in the analysis was 66% as it is now shown in S9 Fig and this result is now presented on Page 11, line 236. Furthermore, we have added the varriant allele frequencies (VAF) of commonly mutated human CRC genes in rhesus CRC as shown in S12 Fig of the supplemental dat. We have also added a comment in the discussion section about these results (Page 16, lines 366-368).*

10. The data in Fig 2 needs backup. The authors should include Suppl material documenting the morphological phenotype(s) of the CRCs in the animals, as well as examples from each IHC reaction to document the interpretation of "absent" and "present." In particular, for LS animals where the assumption is that MLH1 is inactivated by a second hit in the tumor, is there expression of MLH1 and PSM2 in adjacent normal tissue (based on IHC)?

*We agree with Dr. Modiano and thank him for this comment. This comment is also consistent with Reviewer #1 in point #4. Please, refer to our reply for additional information. In brief, in our revised version we now include S4 Fig. This data displays examples of IHC results from an unaffected animal, an MSS phenotype retaining the expression of all MMR proteins and an MSI-H phenotype with loss of expression of MLH1 and PMS2. Six LS animals showed loss of expression of MLH1 and PMS2 proteins; however, IHC results of two LS animals were undetermined (see Fig 2, panel D for full disclosure of the results).*

11. The data do not support the author's conclusions in lines 317 to 321. The leap of faith from the data to these conclusions is far too great.

*We agree with Dr. Modiano and have revised the text to better reflect our interpretation of the CMS data. Furthermore, we add possible reasons why the data differed between human CRC and Rhesus CRC (Page 16, lines 340-353). In brief, one possbiblity may be that tumor stage at diagnosis in rhesus was far more advanced than typically seen in humans where surveillance colonoscopy is standard of care and a very frequent method of diagnosis. Therefore, detection at different stages between humans and rhesus may help explain the CMS differences. Please also see our reply to Reviewer #2, point #1.*

12. The last point is that the authors advance this model recognizing some limitations (of the study), unnecessarily attacking other models (no model is "better" than any other. Each model is imperfect, and all have their own strengths). In criticizing mouse models, for example, the authors fail to acknowledge the exciting advantages and the potential of transposon-driven models. In proposing the Rhesus model for prevention, assessment of TME dynamics (which would require serial invasive interventions), development of therapies, etc., they fail to address the important and considerable limitations of practical animal numbers that would be required for such studies, the time (what is the incidence of the condition in the population and in addition having to support animals in the colony for two decades before tumors develop), the paucity of reagents for NHPs, the costs, and the ethical implications of proposing this grand vision in an era where many people would emphasize reducing animals, and especially primates, used for research. The size of the colony that would be required alone creates a sense of implausibility and makes it difficult to support the rest of the data, which have a place and an impact in helping us understand the role of mutations that affect MMR in the development and progression of CRC.

*We agree with Dr. Modiano and have removed the criticisms to other animal models and the advancing rhetoric of the rhesus model as “superior” or “better” than other animal models. We have extensively edited the text to eliminate those comparisons that we agree were unfair and out of context. For example, we have added the following to the Introduction, “Although all cancer models bear imperfections, the limitations of cellular cultures and murine models warrant the need for different model systems to elucidate the intrinsic and extrinsic factors of MMRd carcinogenesis to help improve clinical outcomes for both LS and MSI sporadic patients” (Page 7, lines 119-122). In addition, the discussion section has been edited accordingly.*

In summary, the Reviewers gave us constructive criticisms and important suggestions to strengthen our manuscript. We feel that the current, revised version of the manuscript is even more improved than our original submission.

Thank you once again for your consideration.

Please do not hesitate to contact me with any questions via email at [evilar@mdanderson.org](mailto:evilar@mdanderson.org)

Sincerely,

Eduardo Vilar, MD, PhD
